# Supplementary material for: A Comparative Study on the Effects of Different Parts of Panax ginseng on the Immune Activity of Cyclophosphamide-Induced Immunosuppressed Mice
Source: Molecules. 2019 Mar 20;24(6):1096. doi: 10.3390/molecules24061096 (PMC6470474; doi:10.3390/molecules24061096)
Supplement: Supplementary file 1 [file molecules-24-01096-s001.pdf]

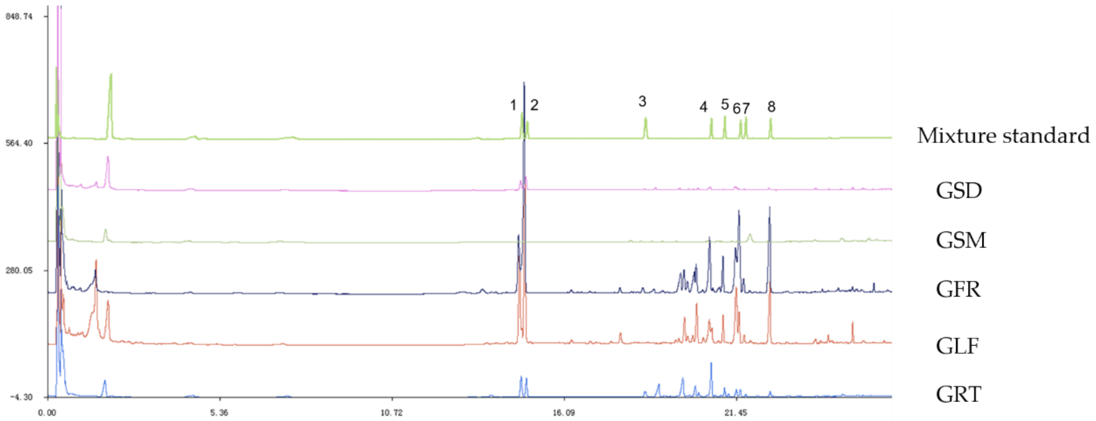

Figure S1. The fingerprint of eight individual ginsenoside in five-year-old ginseng of various parts.

(1: Rg_1; 2: Re; 3: Rf; 4: Rb_1; 5: Rc; 6: Rb_2; 7: Rb_3; 8: Rd).

1. Results

1.1 The content of eight individual ginsenoside in five-year-old ginseng of various parts (Table S1).

The content of ginsenosides has significant difference of various parts ginseng. The total content of eight individual ginsenoside of ginseng were 25.65, 5.35, 89.30, 105.55 and 7.15 mg/g, respectively and all of them had significant difference (P < 0.05).

Table S1 The content of ginsenosides in five-year-old ginseng of various parts

	content of ginsenosides (mg/g)								
Samples	Rg1	Re	Rf	Rb1	Rc	Rb2	Rb3	Rd	Total
GRT	9.05±0.15	4.85±0.13	1.35±0.05	6.15±0.08	1.95±0.12	1.40±0.03	0.20±0.01	0.70±0.01	25.65±0.58^{c}
GSM	1.25±0.07	2.10±0.06	0.35±0.02	0.45±0.01	0.25±0.01	0.45±0.01	0.15±0.01	0.35±0.01	5.35±0.19^{d}
GLF	15.30±0.27	32.55±0.25	2.55±0.08	8.50±0.14	1.70±0.01	10.05±0.18	5.70±0.09	12.95±0.13	89.30±1.15^{b}
GFR	19.00±0.31	39.70±0.29	1.80±0.04	7.35±0.23	8.50±0.25	8.50±0.18	0.90±0.06	19.80±0.34	105.55±1.70^{a}
GSD	1.30±0.05	3.70±0.09	0.33±0.03	0.35±0.01	0.28±0.01	0.50±0.02	0.19±0.02	0.50±0.02	7.15±0.25^{d}

There is no significant difference between the upper standard and the same letter, otherwise there is significant difference (P<0.05)

1.2 The content of seventeen amino acids in five-year-old ginseng of various parts (Table S2).

The content of amino acids of five-year-old ginseng of various parts were 65.77, 50.23, 97.74, 137.53 and 47.96 mg/g, respectively, which had significant difference (P < 0.05).

Table S2 The content of amino acids in five-year-old ginseng of various parts

	The content of amino acids (mg/g)								
samples	Asp	Thr	Ser	Glu	Gly	Ala	Cys	Val	Met
GRT	6.13±0.11	2.18±0.06	1.58±0.02	6.62±0.12	1.42±0.04	4.34±0.09	0.50±0.01	3.07±0.04	0.11±0.00
GSM	6.07±0.17	2.43±0.02	2.41±0.05	8.28±0.12	4.86±0.08	3.52±0.11	0.36±0.01	3.35±0.06	0.20±0.01
GLF	11.29±0.14	5.13±0.09	4.79±0.09	15.65±0.26	10.04±0.16	6.85±0.06	0.49±0.02	6.93±0.13	0.21±0.2
GFR	12.63±0.18	6.37±0.14	5.76±0.08	14.50±0.16	9.30±0.07	11.13±0.12	1.07±0.03	11.05±0.15	0.33±0.01
GSD	6.02±0.14	2.32±0.02	2.48±0.06	7.79±0.10	3.88±0.06	4.13±0.13	0.29±0.01	3.21±0.05	0.11±0.01

	The content of amino acids (mg/g)								
samples	Ile	Leu	Tyr	Phe	Lys	His	Arg	Pro	Total

GRT	4.37±0.09	1.84±0.06	2.43±0.12	2.78±0.04	0.25±0.01	1.39±0.04	21.59±0.12	5.16±0.06	65.77±1.03^{c}
GSM	4.80±0.14	1.72±0.08	2.37±0.06	2.23±0.08	0.28±0.03	1.35±0.07	3.41±0.13	2.60±0.10	50.23±1.32^{d}
GLF	10.11±0.18	3.93±0.07	5.14±0.20	2.97±0.04	0.29±0.02	2.97±0.10	5.83±0.07	5.13±0.07	97.74±1.90^{b}
GFR	15.73±0.16	6.10±0.11	7.56±0.08	4.58±0.09	0.49±0.02	3.45±0.07	18.57±0.37	8.91±0.25	137.53±2.09^{a}
GSD	4.27±0.12	1.43±0.05	2.19±0.03	2.45±0.08	0.25±0.03	1.24±0.06	3.66±0.12	2.24±0.08	47.96±1.15^{d}

There is no significant difference between the upper standard and the same letter, otherwise there is significant difference ($P<0.05$)

1.3 The content of saponins and total polysaccharides in five-year-old ginseng of various parts (Table S3)

The total saponins content of various parts ginseng were 51.39, 23.69, 100.65, 113.78 and 14.12 mg/g, respectively. The trend of saponin content and individual ginsenoside content in different parts of five-year-old ginseng is consistent.

The content of total polysaccharides of various parts ginseng were 22.79, 13.45, 25.09, 31.44 and 29.84 mg/g, respectively.

Table S3 The content of total saponins and total polysaccharide in various parts ginseng

samples	The content of saponins (mg/g)	The content of total polysaccharides (mg/g)
GRT	51.39±2.92^{b}	22.79±2.35^{b}
GSM	23.69±2.70^{c}	13.45±2.22^{c}
GLF	100.65±4.46^{a}	35.09±2.18^{a}
GFR	113.78±4.19^{a}	31.44±2.41^{a}
GSD	14.12±0.12^{d}	29.84±2.16^{a}

There is no significant difference between the upper standard and the same letter, otherwise there is significant difference ($P<0.05$)
